# Widespread alterations in white matter microstructure in autism: A multilevel meta-analysis

**DOI:** 10.21203/rs.3.rs-7411531/v1

**Published:** 2025-09-23

**Authors:** Christy D. Yoon, Andrew L. Alexander, Brittany G. Travers, Janet E. Lainhart, Douglas C. Dean

**Affiliations:** Waisman Center, University of Wisconsin-Madison, Madison, WI, USA; Waisman Center, University of Wisconsin-Madison, Madison, WI, USA; Department of Medical Physics, University of Wisconsin-Madison, Madison, WI, USA; Department of Psychiatry, University of Wisconsin-Madison, Madison, WI, USA; Waisman Center, University of Wisconsin-Madison, Madison, WI, USA; Department of Kinesiology, Occupational Therapy Program, University of Wisconsin-Madison, Madison, WI, USA; Waisman Center, University of Wisconsin-Madison, Madison, WI, USA; Department of Psychiatry, University of Wisconsin-Madison, Madison, WI, USA; Waisman Center, University of Wisconsin-Madison, Madison, WI, USA; Department of Medical Physics, University of Wisconsin-Madison, Madison, WI, USA; Department of Pediatrics, University of Wisconsin-Madison, Madison, WI, USA

**Keywords:** autism, DTI, meta-analysis, microstructure, quantitative MRI, white matter

## Abstract

**Background:**

Disrupted brain connectivity is central to understanding the neurobiological basis of autism spectrum disorder (ASD). Diffusion tensor imaging (DTI) has been extensively used to study brain microstructure in ASD, which often reveals reduced fractional anisotropy (FA) and increased mean diffusivity (MD), indicating altered microstructural integrity.

**Methods:**

We conducted a multivariate random-effects meta-analysis with a multilevel structure to evaluate the extent to which FA and MD measures of white matter microstructure are altered in individuals with ASD compared to typically developing (TD) individuals, as well as how participant characteristics and laterality moderate the magnitude of the estimated differences. Our analysis included 680 effect sizes from 59 studies (*N*_*ASD*_ = 1,750, *N*_*TD*_ = 1,484; *M*_*age*_ = 2–50 years) across 11 white matter tracts.

**Results:**

We found a significant moderate summary effect size for FA in eight tracts (corpus callosum, corticospinal tract, thalamic radiation, arcuate fasciculus, inferior fronto-occipital fasciculus [IFOF], inferior longitudinal fasciculus [ILF], superior longitudinal fasciculus [SLF], and uncinate fasciculus; Hedges’ *g* = −0.40 to −0.59), suggesting that individuals with ASD have lower FA values in these tracts compared to TD controls. Additionally, we found a significant moderate-to-large summary effect size for MD in six tracts (corpus callosum, corona radiata, arcuate fasciculus, IFOF, ILF, and SLF; Hedges’ *g* = 0.32 to 1.09), suggesting that individuals with ASD have higher MD values in these tracts compared to TD controls. Furthermore, moderators demonstrated tract- and metric-specific effects.

**Limitations:**

Participants were primarily male, and the age range excludes later adulthood when white matter deterioration becomes more evident. Therefore, our findings are limited in generalizing to females and may not be applicable across the entire lifespan.

**Conclusions:**

Collectively, our findings highlight the complex and multidimensional nature of white matter alterations observed in ASD.

## Background

Since the early 2000s, theories of disrupted brain connectivity have been central to explaining the neurobiological basis of autism spectrum disorder (ASD). These models propose that ASD is characterized by reduced long-range connectivity, particularly between the frontal and posterior cortical regions, along with heightened local connectivity within specific networks [1, 2, 3, 4]. It is hypothesized that these patterns reflect widespread disturbances in the development and coordination of large-scale neural systems, potentially contributing to the cognitive and core behavioral features of ASD. Such connectivity-based accounts have inspired a growing body of neuroimaging research aimed at empirically characterizing atypical patterns of brain organization in ASD.

Supporting this claim, functional magnetic resonance imaging (fMRI) studies have identified atypical synchrony in large-scale brain networks among individuals with ASD [5, 6, 7]. However, while fMRI provides valuable insights into functional brain dynamics, it offers indirect measurements of neural activity by capturing the blood-oxygen-level-dependent signal, which reflects vascular changes that correlate with, but do not directly indicate, neuronal firing [8]. Additionally, these hemodynamic signals are influenced by transient mental states [9] and motion artifacts [10]. Importantly, functional connectivity does not necessarily depict the anatomical pathways underlying interregional communication. Therefore, functional findings alone provide a limited view of the neural architecture underlying ASD.

This recognition has fueled a growing interest in structural imaging methods, particularly diffusion tensor imaging (DTI) [11], which has become an important tool for investigating brain connectivity in ASD. DTI enables in vivo quantitative assessment of white matter microstructure and provides complementary insights into the anatomical substrates that support interregional communication. It generates key metrics, including fractional anisotropy (FA), mean diffusivity (MD), radial diffusivity (RD), and axial diffusivity (AD) [11], which are expected to reflect underlying features such as fiber coherence, myelination, and axonal density. Differences in these measures in ASD have been reported across various white matter tracts involved in social, language, and executive functioning, including the corpus callosum [12, 13, 14, 15, 16], arcuate fasciculus [15, 17], inferior and superior longitudinal fasciculi [18, 19], uncinate fasciculus [17, 18], cingulum bundle [15, 18], as well as thalamocortical [20] and fronto-occipital pathways [19, 21]. These differences often manifest as reduced FA and increased MD, indicating altered microstructural integrity in these pathways. Studies also highlight region-specific findings, such as altered hemispheric asymmetry [14], reduced coherence of white matter microstructure [13], and associations with ASD feature prominence [19, 21]. However, despite the expansion of the DTI literature, findings remain inconsistent, with some studies reporting widespread white matter disruptions while others identify more regionally specific or developmentally variable differences, including limited or nonsignificant differences [15, 17, 18]. This variability has prompted clarification regarding the consistency and specificity of white matter differences in ASD.

Several meta-analyses have been conducted to quantitatively synthesize DTI findings and assess the reliability and scope of reported differences in ASD. Aoki et al. (2013) [22] conducted the first meta-analysis of DTI studies to evaluate tract-specific differences in ASD. They reported reduced FA in the corpus callosum, superior longitudinal fasciculus (SLF), and uncinate fasciculus, alongside increased MD in the corpus callosum and SLF in individuals with ASD compared to typically developing (TD) individuals. However, Aoki et al. primarily focused on FA, providing more limited treatment of MD across a restricted number of white matter tracts, and potential moderating variables, such as age or sex, that could contribute to heterogeneity in effect sizes were left unexamined. More recent meta-analyses of DTI studies have adopted narrower or transdiagnostic scopes. For instance, Li et al. (2022) [23] specifically focused on language-related white matter pathways and found consistent disruptions in dorsal and ventral tracts, particularly in the left hemisphere and among children compared to adults. While informative for understanding language impairments, global white matter organization or tracts outside the language network were not evaluated. Other meta-analyses [24, 25] employed transdiagnostic frameworks to examine white matter differences across ASD, attention-deficit/hyperactivity disorder, and other neurodevelopmental disorders. While the two meta-analyses revealed shared alterations [24, 25], notably in the corpus callosum, they were not intended to isolate ASD-specific profiles. Collectively, while previous meta-analyses have advanced our understanding of white matter alterations in ASD, most remain constrained by limited tract coverage, a focus on specific domains, or the inclusion of heterogeneous diagnostic groups.

Therefore, this meta-analysis aims to address these gaps by providing a comprehensive synthesis of quantitative neuroimaging studies in ASD that encompass extensive white matter tracts and clarify the anatomical distribution and robustness of white matter alterations associated with ASD. Meta-analytic techniques offer a robust statistical framework for evaluating the consistency, magnitude, and generalizability of observed effects, while also exploring the influence of potential moderating variables. Accordingly, this work aims to (1) quantify summary effect sizes for group differences between individuals with ASD and TD individuals across a wide range of white matter tracts, and (2) assess how factors such as participant characteristics (e.g., age, sex, and IQ) and laterality may contribute to variability in effect sizes.

## Methods

We conducted our meta-analysis in accordance with the Preferred Reporting Items for Systematic Reviews and Meta-Analyses Statement (PRISMA) [26] guideline.

### Study Selection

We utilized two sources to identify relevant articles. First, we conducted a search in January 2025 across four databases—MEDLINE, PsycArticles, PsycINFO, and PubMed—using search parameters aligned with our areas of interest: (a) autism (“*autism*” OR “*ASD*” OR “*autism spectrum disorder*” OR “autis*”); and (b) MRI (“*diffusion MRI*” OR “*constrained spherical deconvolution*” OR “*CSD*” OR “*diffusion kurtosis imaging*” OR “*DKI*” OR “diffusion tensor *imaging*” OR “*DTI*” OR “*neurite orientation dispersion and density imaging*” OR “*NODDI*” OR “*relaxometry*” OR “*quantitative MRI*” OR “*T1 mapping*” OR “*T2 mapping*”). Second, we included studies from three recent meta-analyses on DTI in ASD [23, 24, 25] to identify studies that may have been overlooked in the database searches.

We applied the following eligibility criteria: Articles must (a) be published in a peer-reviewed journal, be empirical, and be written in English; (b) include ASD and TD control groups; (c) apply a quantitative MRI technique (e.g., CSD, DKI, DTI, NODDI, relaxometry); (d) derive a quantitative microstructural metric (e.g., FA, MD, RD, AD) from a white matter tract; and (e) report a between-group effect size (Cohen’s *d*) or other statistics (e.g., means, standard deviation [SD], standard error [SE], test statistics) to calculate differences in white matter microstructure between the ASD and TD groups. There were no restrictions on publication date or participant age.

**Figure 1:**
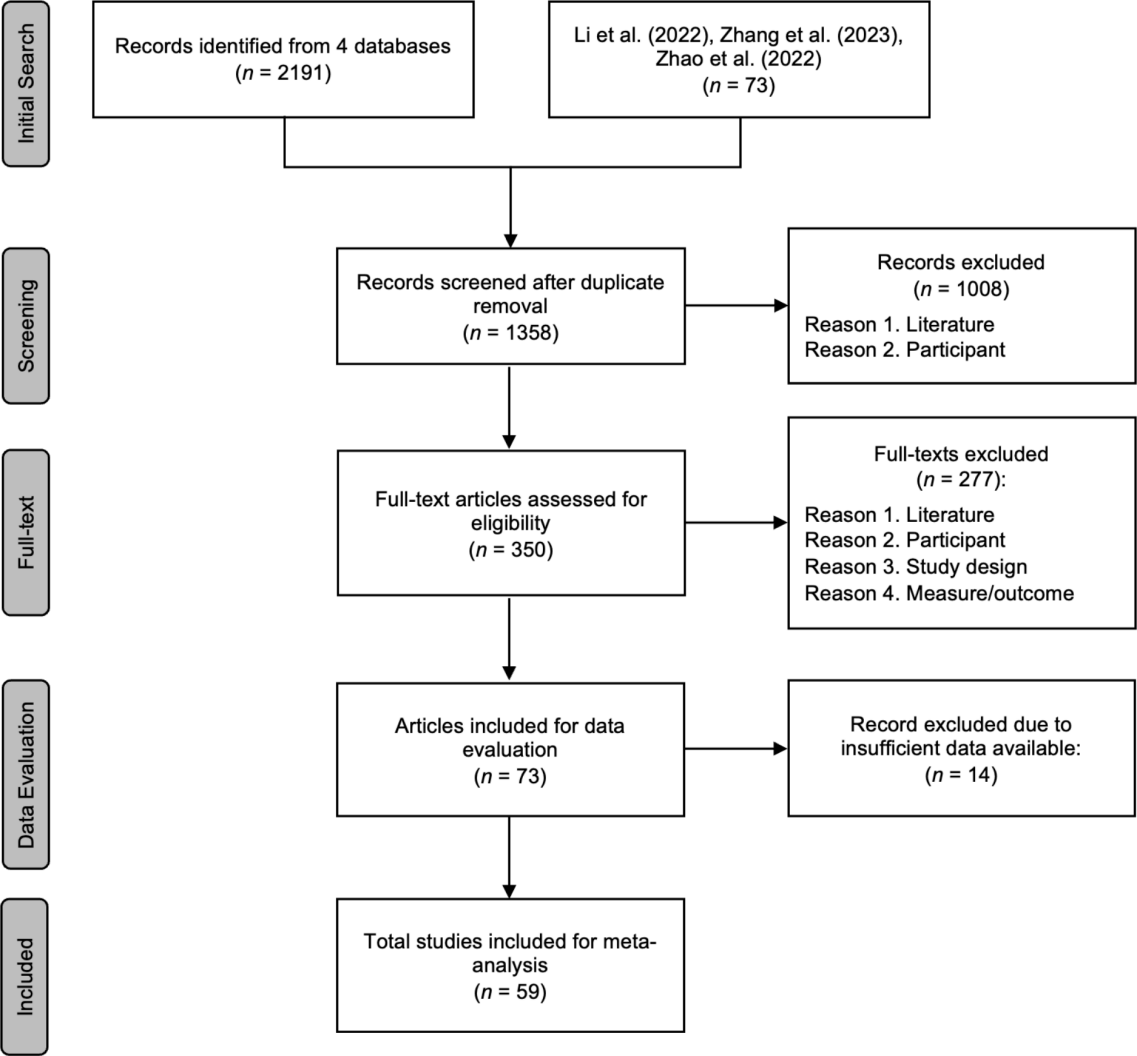
PRISMA flow chart

The initial search yielded a total of 2,264 articles. After removing duplicates, 1,358 articles remained for screening. These articles were assessed against the eligibility criteria in two stages. First, the titles and abstracts of the 1,358 articles were screened according to the eligibility criteria, resulting in the exclusion of 1,008 articles. Subsequently, the full texts of the remaining 350 articles were reviewed, leading to the removal of 277 articles. Consequently, 73 articles were included for data evaluation. See Fig. 1 for PRISMA flow chart for study selection process.

### Data Evaluation

We extracted effect sizes (Cohen’s *d*) that indicate differences in white matter microstructure between the ASD and TD groups. When effect sizes were not reported, we extracted available information (e.g., sample sizes, means, SD, SE, and test statistics) to calculate Cohen’s *d*. For each effect size, we also extracted data on participant age in years (mean, SD), sex (proportion of male participants), and full-scale IQ (mean, SD) for both the ASD and TD groups, as well as laterality (left or right), when available. To ensure consistent interpretation of effect sizes, we revised their signs where necessary (i.e., a negative effect size indicates lower values for the ASD group, while a positive effect size indicates higher values for the ASD group), aligning all effect sizes uniformly. Many studies reported multiple relevant effect sizes, including those from tract subregions, such as the corpus callosum, or from the left and right hemisphere. Consequently, the data were inherently hierarchical, with estimates of group differences nested within studies. Each study and its unique effect sizes were coded to account for this multilevel structure. All reported and calculated Cohen’s *d* values were converted to Hedges’ *g*, applying a correction factor to provide an unbiased estimate of the standardized mean difference [27]. Notably, while our meta-analysis initially sought to incorporate various quantitative MRI techniques, such as CSD, DKI, DTI, NODDI, and relaxometry (i.e., eligibility criteria (c)), the final analysis focused exclusively on DTI (specifically FA and MD metrics) due to inadequate effect sizes or information necessary to compute those from studies using techniques other than DTI, along with limited effect sizes that contributed to AD and RD metrics of DTI. As a result, 14 studies were removed during data evaluation, and a total of 59 studies were included in the final analysis. See Table S1 for the references of the studies included.

### Quality Assessment

The quality of 59 studies was assessed based on selection, comparability, and outcome using a modified version of the Newcastle-Ottawa Quality Assessment Scale [28]. An additional criterion was included to determine whether studies controlled for head motion, due to its known impact on diffusion measures (Table S1).

### Statistical Analysis

#### Model Specification for Meta-analysis

We conducted a multivariate random-effects meta-analysis with a three-level structure [29, 30] using the ‘*metafor’* package [31]. This approach accounts for the nested data structure, where multiple effect sizes are reported per study, by partitioning variance into sampling, within-study, and between-study components, as well as incorporating a variance-covariance matrix to model the correlation between effect sizes within studies. To assess the robustness of the assumed intra-study effect size correlation, we performed sensitivity analyses under varying assumptions of rho (*ρ* = 0.3, 0.6, and 0.9) and compared changes in model estimates. This approach allows us to appropriately model statistical dependence and test the stability of summary effect sizes under different assumptions. Notably, across all tracts, sensitivity analyses indicated minimal changes in summary effect sizes as *ρ* varied from 0.3 to 0.9, with consistent magnitude classification and statistical inference, suggesting stability across dependency structures. Therefore, we report all analyses using a *ρ* value of 0.6, a midpoint that reflects the observed stability across the range. See Table S2 for full sensitivity analysis results.

#### Bias and Influential Effects

To evaluate potential sources of bias and assess the robustness of the meta-analytic findings, we conducted a series of diagnostic and sensitivity analyses. First, we included the SE of each effect size as a predictor in the multilevel meta-analytic model [32]. This allows us to examine whether studies with greater sampling variability tend to report systematically different effect sizes while accounting for the statistical dependency of multiple effect sizes nested within studies. We also visually inspected funnel plots for asymmetry [33], which may indicate the presence of small-study effects or publication bias. Finally, we utilized Cook’s Distance [34] to identify influential effect sizes that had a disproportionate impact on the model estimates, aiding in the detection of potential outliers or high-leverage points. Where applicable, we conducted sensitivity analyses (*ρ* = 0.3, 0.6, and 0.9) by re-estimating the model after removing influential cases to evaluate the stability of the re-estimated summary effect size.

#### Moderation Analysis

After estimating the summary effect sizes and conducting bias diagnostics, we performed meta-regression analyses using the same multivariate three-level model specification to determine whether variations in effect sizes could be explained by study-level characteristics. We assessed age (in years), sex (% male), full-scale IQ, and laterality (left, right), each in separate models. This approach maximizes statistical power, particularly given the incomplete reporting across studies, and allows us to identify the unique contribution of each variable to the variation in effect sizes [29, 30, 31]. Meta-regression was conducted regardless of heterogeneity (i.e., *I*^*2*^ > 75%) [35] or statistical significance to minimize model misspecification, explore potential moderator effects, and account for residual between-study variance. See Table S3 for an explanation of the moderator selection.

## Results

**Figure 2:**
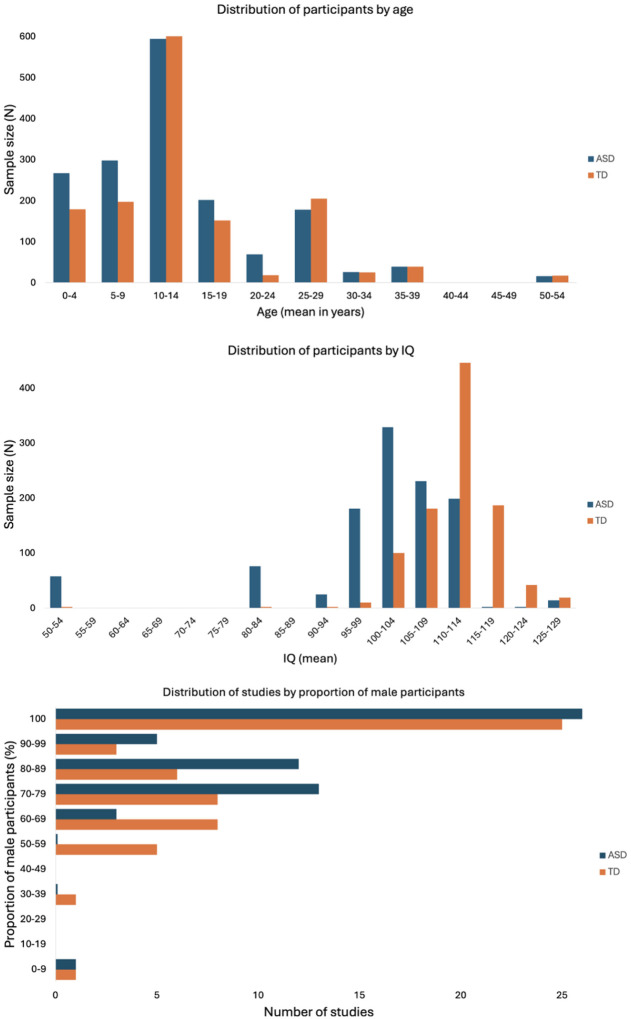
Distribution of participant characteristics (age, sex, and IQ).

**Figure 3:**
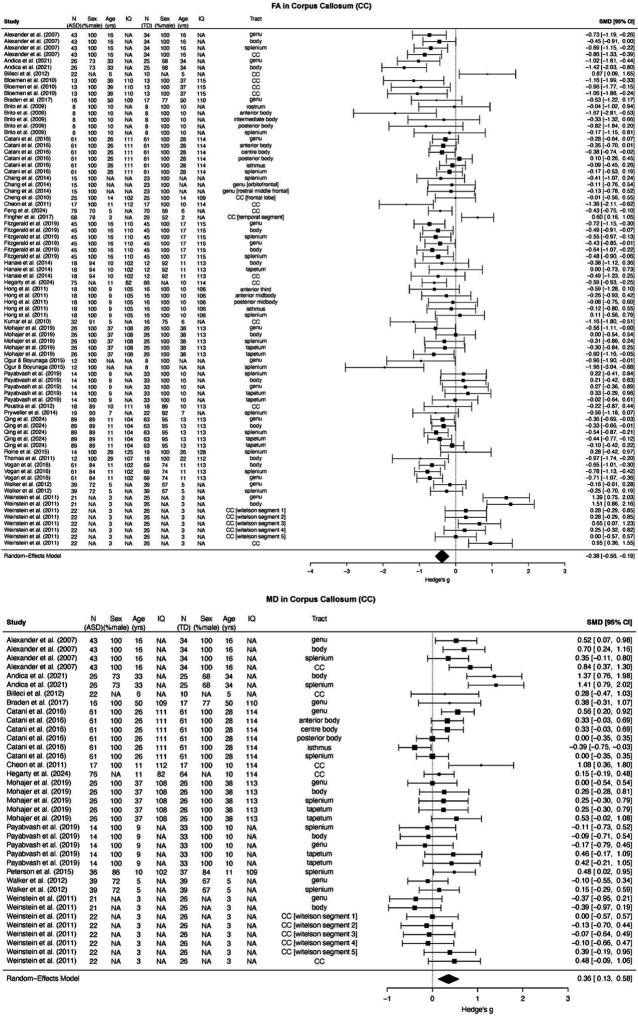
Forest plots for the corpus callosum.

The analysis included 680 effect sizes (*n* = 59 studies) that indicated FA and MD measures of microstructural differences between the ASD and TD groups across 11 white matter tracts. The final dataset comprised 3,234 participants (*N*_*ASD*_ = 1,750, *N*_*TD*_ = 1,484), with an average age ranging from 2 to 50 years (Fig. 2). Details regarding each effect size are provided in Fig. 3 and Figure S1. Results are organized by commissural, projection, and association tracts.

### Commissural Tract

#### Corpus Callosum

The corpus callosum included 79 effect sizes for FA (*N*_*ASD*_ = 907, *N*_*TD*_ = 830) and 37 effect sizes for MD (*N*_*ASD*_ = 398, *N*_*TD*_ = 391), encompassing the whole and its subregions: the genu, body, splenium, isthmus, rostrum, and tapetum.

##### Group differences.

**Figure 4:**
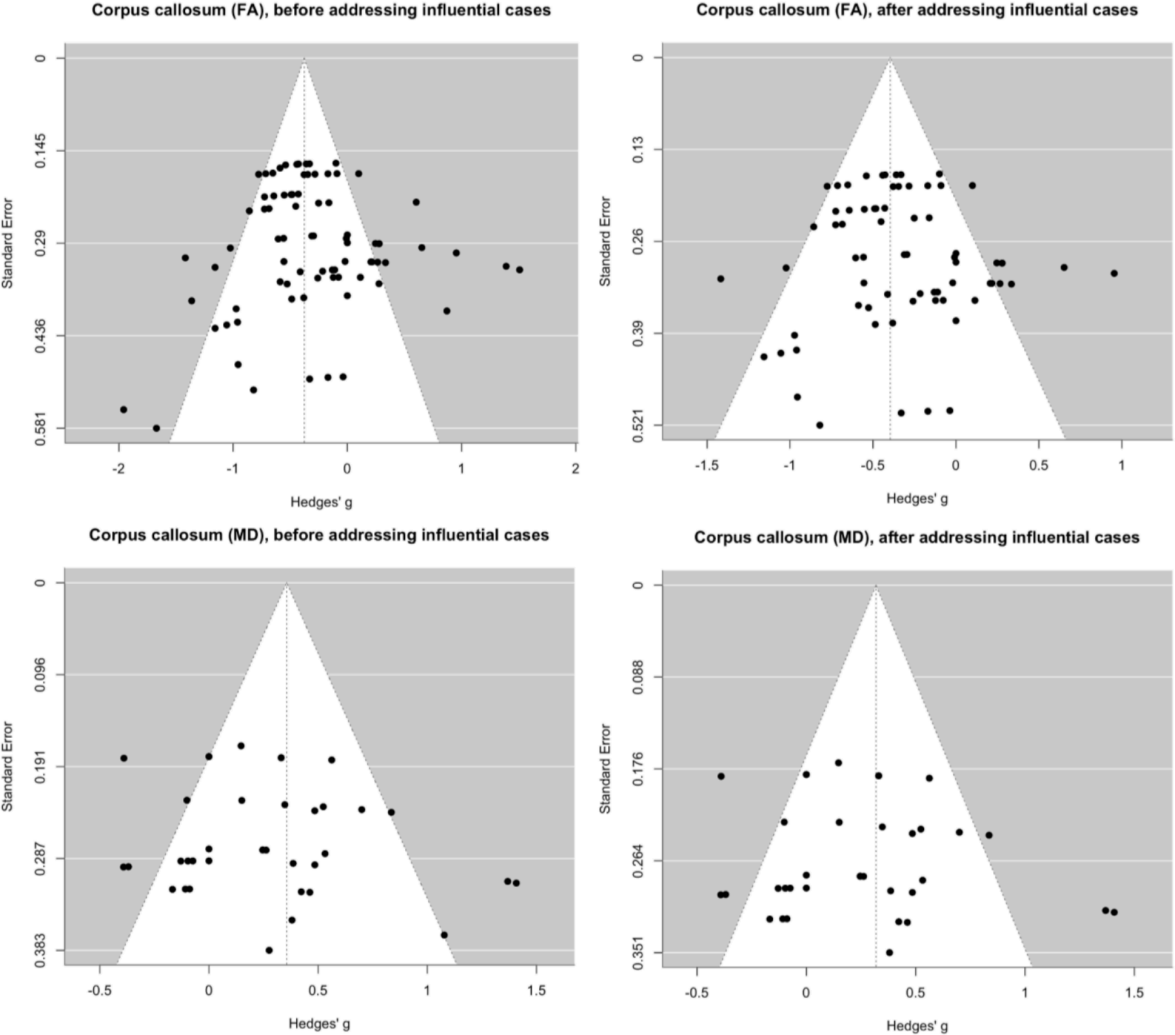
Funnel plots for the corpus callosum.

The analysis revealed a significant moderate summary effect size for both FA and MD in the corpus callosum (FA: *g* = −0.38, 95% CI [−0.56, −0.19], *p* < 0.001; MD: *g* = 0.36, 95% CI [0.13, 0.58], *p* = 0.002; Fig. 3), indicating lower FA and higher MD in the corpus callosum in ASD compared to TD. There was no evidence of small-study effects (FA: *b* = −0.89, 95% CI [−2.90, 1.12], *p* = 0.387; MD: *b* = 2.39, 95% CI [−1.11, 5.88], *p* = 0.181) or notable asymmetry in the funnel plots (Fig. 4); however, several influential effect sizes were identified (*k*_*FA*_ = 10, *k*_*MD*_ = 2; Figure S3(a)). When re-estimating the summary effect sizes without these influential cases, the results remained moderate and significant for both FA (*g* = −0.40, *p* < 0.001) and MD (*g* = 0.32, *p* = 0.008) in the corpus callosum.

##### Moderating Effects.

FA in the corpus callosum was significantly moderated by age (*b* = −0.02, *p* = 0.024), suggesting that differences in FA in the corpus callosum between ASD and TD are greater at older ages. In contrast, MD in the corpus callosum was not moderated by age. IQ did not moderate FA in the corpus callosum, and its effect on MD in the corpus callosum could not be assessed due to insufficient available data. Neither summary effect size was moderated by sex, and laterality was not assessed due to its inapplicability to the corpus callosum. See Table 1 for a summary of the results.

[See the attached file for Fig. 3.]

#### Projection Tracts

The projection tracts comprised five distinct tracts: the cerebellar peduncles, corona radiata, corticospinal tract, internal capsule, and thalamic radiation. See Figures S1(a)-(e), S2(a)-(e), and S3(b)-(f) for forest plots, funnel plots, and Cook’s distance for projection tracts.

#### Cerebellar Peduncles

The cerebellar peduncles included 36 effect sizes for FA (*N*_*ASD*_ = 255, *N*_*TD*_ = 218) and 15 effect sizes for MD (*N*_*ASD*_ = 201, *N*_*TD*_ = 187), encompassing the whole, inferior, middle, and superior cerebellar peduncles.

##### Group differences.

Neither FA nor MD in the cerebellar peduncles showed a significant summary effect size (FA: *g* = −0.04, 95% CI [−0.48, 0.40], *p* = 0.862; MD: *g* = 0.53, 95% CI [−0.75, 1.81], *p* = 0.417). There was no evidence of small-study effects (*b* = −3.13, 95% CI [−7.35, 1.09], *p* = 0.146) or notable asymmetry in the funnel plot for FA; however, small-study bias was evident (*b* = 12.68, 95% CI [9.26, 16.09], *p* < 0.001), with considerable funnel plot asymmetry for MD. Influential effect sizes were also identified (*k*_*FA*_ = 4, *k*_*MD*_ = 2). The re-estimated summary effect sizes remained consistent in magnitude classification and statistical inference for both FA (*g* = 0.08, *p* = 0.731) and MD (*g* = 0.70, *p* = 0.429) in the cerebellar peduncles. However, the direction of the summary effect size for FA shifted from negative to positive, indicating potential sensitivity to specific studies and possible instability, which warrants caution when interpreting subsequent moderator effect results for FA in the cerebellar peduncles.

##### Moderating effects.

Neither FA nor MD in the cerebellar peduncles was moderated by age, sex, or laterality. IQ could not be assessed due to insufficient data available (Table 1).

#### Corona Radiata

The corona radiata included 35 effect sizes for FA (*N*_*ASD*_ = 386, *N*_*TD*_ = 379) and 17 effect sizes for MD (*N*_*ASD*_ = 171, *N*_*TD*_ = 176), encompassing the whole, anterior, posterior, and superior corona radiata.

##### Group differences.

The summary effect size for FA in the corona radiata was non-significant (*g* = −0.14, 95% CI [−0.47, 0.19], *p* = 0.416). Conversely, a significant moderate summary effect size was observed for MD in the corona radiata (*g* = 0.29, 95% CI [0.01, 0.56], *p* = 0.040), indicating higher MD in the corona radiata in ASD compared to TD. There was no evidence of small-study effects (FA: *b* = 1.90, 95% CI [−1.91, 5.71], *p* = 0.329; MD: *b* = −1.37, 95% CI [−4.40, 1.66], *p* = 0.376) or notable funnel plot asymmetry; however, several influential effect sizes were identified (*k*_*FA*_ = 2, *k*_*MD*_ = 2). The re-estimated summary effect sizes remained consistent in magnitude classification and statistical inference for both FA (*g* = −0.12, *p* = 0.494) and MD (*g* = 0.34, *p* < 0.001) in the corona radiata.

##### Moderating effects.

Both FA and MD in the corona radiata was significantly moderated by sex (FA: *b* = 0.04, *p* = 0.019; MD: *b* = −0.04, *p* = 0.024), suggesting that differences in FA and MD in the corona radiata between ASD and TD decrease as the proportion of male participants increases. Neither summary effect size was moderated by age, IQ, or laterality (Table 1).

#### Corticospinal Tract

The corticospinal tract included 34 effect sizes for FA (*N*_*ASD*_ = 469, *N*_*TD*_ = 357) and 15 effect sizes for MD (*N*_*ASD*_ = 168, *N*_*TD*_ = 142), encompassing the whole, inferior, middle, and superior corticospinal tract.

##### Group differences.

A significant moderate summary effect size was found for FA in the corticospinal tract (*g* = −0.44, 95% CI [−0.73, −0.14], *p* = 0.004), indicating lower FA in the corticospinal tract in ASD compared to TD. Meanwhile, a non-significant summary effect size was revealed for MD in the corticospinal tract (*g* = 0.75, 95% CI [−0.06, 1.55], *p* = 0.070). There was no evidence of small-study effects (*b* = −1.98, 95% CI [−5.13, 1.17], *p* = 0.217) or notable asymmetry in the funnel plot for FA; however, for MD, small-study effects (*b* = 17.12, 95% CI [12.90, 21.35], *p* < 0.001) and notable funnel plot asymmetry were evident. Several influential effect sizes were also identified (*k*_*FA*_ = 4, *k*_*MD*_ = 3). The re-estimated summary effect sizes remained consistent in both magnitude classification and statistical inferences for both FA (*g* = −0.47, *p* = 0.015) and MD (*g* = 1.01, *p* = 0.111) in the corticospinal tract.

##### Moderating effects.

FA in the corticospinal tract was significantly moderated by IQ (*b* = 0.03, *p* = 0.039), suggesting that differences in FA in the corticospinal tract between ASD and TD decrease as IQ increases. In contrast, MD in the corticospinal tract was not moderated by IQ. Neither summary effect size was moderated by age, sex, or laterality (Table 1).

#### Internal Capsule

The internal capsule included 30 effect sizes for FA (*N*_*ASD*_ = 354, *N*_*TD*_ = 326) and 13 effect sizes for MD (*N*_*ASD*_ = 225, *N*_*TD*_ = 202), encompassing the whole, anterior limb, posterior limb, and retrolenticular part.

##### Group differences.

The analysis revealed non-significant summary effect sizes for both FA and MD in the internal capsule (FA: *g* = −0.32, 95% CI [−0.66, 0.02], *p* = 0.066; MD: *g* = 0.32, 95% CI [−0.10, 0.74], *p* = 0.135). There was no evidence of small-study effects (FA: *b* = −2.16, 95% CI [−5.11, 0.80], *p* = 0.152; MD: *b* = 6.79, 95% CI [−0.60, 14.18], *p* = 0.072) or notable asymmetry in the funnel plots; however, several influential effect sizes were identified (*k*_*FA*_ = 3, *k*_*MD*_ = 1). The re-estimated summary effect sizes stayed moderate and non-significant for both FA (*g* = −0.21, *p* = 0.306) and MD (*g* = 0.31, *p* = 0.275) in the internal capsule.

##### Moderating effects.

Neither FA nor MD in the internal capsule was moderated by age, sex, or laterality. IQ could not be assessed due to insufficient data availability (Table 1).

#### Thalamic Radiation

The thalamic radiation included 38 effect sizes for FA (*N*_*ASD*_ = 584, *N*_*TD*_ = 494) and 19 effect sizes for MD (*N*_*ASD*_ = 293, *N*_*TD*_ = 232), encompassing anterior, posterior, and superior thalamic radiation.

##### Group differences.

A significant moderate summary effect size was found for FA in the thalamic radiation (*g* = −0.42, 95% CI [−0.72, −0.11], *p* = 0.007), indicating lower FA in the thalamic radiation in ASD compared to TD. Conversely, a non-significant summary effect size was observed for MD in the thalamic radiation (*g* = 0.20, 95% CI [−0.06, 0.46], *p* = 0.139). There was no evidence of small-study effects (FA: *b* = −2.85, 95% CI [−6.47, 0.77], *p* = 0.122; MD: *b* = 0.71, 95% CI [−4.48, 5.91], *p* = 0.787) or notable asymmetry in funnel plots; however, several influential effect sizes were identified (*k*_*FA*_ = 3, *k*_*MD*_ = 1). The re-estimated summary effect sizes remained consistent in both magnitude classification and statistical inference for both FA (*g* = −0.46, *p* < 0.001) and MD (*g* = 0.08, *p* = 0.382) in the thalamic radiation.

##### Moderating effects.

FA in the thalamic radiation was significantly moderated by sex (*b* = 0.01, *p* = 0.041) and IQ (*b* = 0.05, *p* = 0.002), suggesting that differences in FA in the thalamic radiation between ASD and TD are less pronounced with a larger proportion of male participants or a higher IQ. In contrast, MD in the thalamic radiation was not moderated by sex or IQ. Neither summary effect size was moderated by age or laterality (Table 1).

#### Association Tracts

The association tracts comprised five distinct tracts: arcuate fasciculus, inferior fronto-occipital fasciculus (IFOF), inferior longitudinal fasciculus (ILF), superior longitudinal fasciculus (SLF), and uncinate fasciculus. See Figures S1(f)-(j), S2(f)-(j), and S3(g)-(k) for forest plots, funnel plots, and Cook’s distance for association tracts.

#### Arcuate Fasciculus

The arcuate fasciculus included 40 effect sizes for FA (*N*_*ASD*_ = 302, *N*_*TD*_ = 253) and 18 effect sizes for MD (*N*_*ASD*_ = 127, *N*_*TD*_ = 106), encompassing the whole, anterior, long, and posterior arcuate fasciculus.

##### Group Differences.

The analysis revealed a significant moderate summary effect size for FA in the arcuate fasciculus (*g* = −0.35, 95% CI [−0.60, −0.10], *p* = 0.006) and a significant large summary effect size for MD in the arcuate fasciculus (*g* = 0.98, 95% CI [0.32, 1.63], *p* = 0.004). This indicates lower FA and notably higher MD in the arcuate fasciculus in ASD compared to TD. The bias assessment showed no evidence of small-study effects (*b* = −2.02, 95% CI [−4.45, 0.41], *p* = 0.103) or a notable asymmetric funnel plot for FA; however, it revealed small-study effects (*b* = 12.52, 95% CI [8.73, 16.31], *p* < 0.001) and a notable asymmetric funnel plot for MD. Several influential effect sizes were also identified (*k*_*FA*_ = 8, *k*_*MD*_ = 1). The re-estimated summary effect sizes remained consistent in both magnitude classification and significance for both FA (*g* = −0.40, *p* = 0.008) and MD (*g* = 1.09, *p* = 0.010) in the arcuate fasciculus.

##### Moderating Effects.

Both FA and MD in the arcuate fasciculus was significantly moderated by sex (FA: *b* = 0.02, *p* = 0.051; MD: *b* = −0.17, *p* < 0.001), suggesting that differences in FA and MD in the arcuate fasciculus between ASD and TD decrease as the proportion of male participants increases. Neither summary effect size was moderated by age or laterality. IQ was not assessed due to insufficient available data (Table 1).

#### Inferior Fronto-occipital Fasciculus

The IFOF included 35 effect sizes for FA (*N*_*ASD*_ = 370, *N*_*TD*_ = 348) and 13 effect sizes for MD (*N*_*ASD*_ = 190, *N*_*TD*_ = 204), encompassing the whole, anterior, and posterior IFOF.

##### Group differences.

A significant moderate summary effect size for FA in IFOF (*g* = −0.54, 95% CI [−0.98, −0.10], *p* = 0.016) and a significant moderate-to-large summary effect size for MD in IFOF were identified (*g* = 0.73, 95% CI [0.47, 1.00], *p* < 0.001). This indicates lower FA and notably higher MD in IFOF in ASD compared to TD. For FA, small-study effects were evident (*b* = −5.59, 95% CI [−9.07, −2.11], *p* = 0.002), along with an asymmetric funnel plot and influential effect sizes (*k*_*FA*_ = 3). However, for MD, there was no evidence of small-study effects (*b* = −0.40, 95% CI [−3.60, 2.81], *p* = 0.809), asymmetry in the funnel plot, or influential effect size, indicating that the original estimate for MD in IFOF is stable and robust. Re-estimating the FA model increased its summary effect size (*g* = −0.59, *p* = 0.007), which remained moderate and significant.

##### Moderating effects.

FA in IFOF was significantly moderated by age (*b* = 0.06, *p* = 0.013) and IQ (*b* = 0.09, *p* < 0.001), suggesting that differences in FA in IFOF between ASD and TD are smaller at older ages or with higher IQ. However, neither age nor IQ moderated MD in IFOF. Conversely, sex significantly moderated MD in IFOF (*b* = −0.02, *p* = 0.034), suggesting that group differences in MD in IFOF are smaller with a higher proportion of male participants, while it did not moderate FA in IFOF. Notably, FA in IFOF was significantly moderated by laterality (*b*_*right*_ = 0.20, *p* = 0.012), suggesting that group differences are smaller in the right IFOF compared to the left IFOF, whereas laterality did not moderate MD in IFOF (Table 1).

#### Inferior Longitudinal Fasciculus

The ILF included 34 effect sizes for FA (*N*_*ASD*_ = 492, *N*_*TD*_ = 484) and 13 effect sizes for MD (*N*_*ASD*_ = 207, *N*_*TD*_ = 215).

##### Group differences.

A significant moderate summary effect size for both FA and MD in ILF was revealed (FA: *g* = −0.56, 95% CI [−0.90, −0.23], *p* < 0.001; MD: *g* = 0.51, 95% CI [0.15, 0.87], *p* = 0.005), indicating lower FA and higher MD in ILF in ASD compared to TD. Evidence of small-study effects was observed for FA (*b* = −3.25, 95% CI [−6.38, −0.11], *p* = 0.042), along with funnel plot asymmetry, whereas no such evidence was found for MD (*b* = −2.97, 95% CI [−6.80, 0.86], *p* = 0.129), with no notable asymmetry in its funnel plot. However, several influential effect sizes were identified (*k*_*FA*_ = 3, *k*_*MD*_ = 2). The re-estimated summary effect sizes remained moderate and significant for both FA (*g* = −0.49, *p* = 0.004) and MD (*g* = 0.55, *p* = 0.002) in ILF.

##### Moderating effects.

FA in ILF was significantly moderated by age (*b* = 0.04, *p* = 0.007) and IQ (*b* = 0.07, *p* < 0.001), suggesting that differences in FA in ILF between ASD and TD are smaller at older ages or with higher IQ. However, these factors did not moderate MD in ILF. Neither summary effect size was moderated by sex or laterality (Table 1).

#### Superior Longitudinal Fasciculus

The SLF included 66 effect sizes for FA (*N*_*ASD*_ = 623, *N*_*TD*_ = 564) and 26 effect sizes for MD (*N*_*ASD*_ = 328, *N*_*TD*_ = 343).

##### Group differences.

A non-significant summary effect size was found for FA in SLF (*g* = −0.24, 95% CI [−0.57, 0.10], *p* = 0.166). In contrast, a significant moderate summary effect size for MD in SLF was identified (*g* = 0.69, 95% CI [0.22, 1.15], *p* = 0.004), indicating higher MD in SLF in ASD compared to TD. Evidence of small-study effects was present (FA: *b* = −8.63, 95% CI [−11.27, −5.98], *p* < 0.001; MD: *b* = 7.28, 95% CI [4.03, 10.52], *p* < 0.001), along with asymmetric funnel plots and several influential effect sizes (*k*_*FA*_ = 7, *k*_*MD*_ = 7). The re-estimated summary effect size for FA in SLF remained moderate but became significant (*g* = −0.40, *p* = 0.027), suggesting lower FA in SLF in ASD compared to TD, while it remained moderate and significant for MD in SLF (*g* = 0.66, *p* = 0.003). Although addressing influential cases appeared to improve the summary effect size for FA, the change from non-significant to significant may reflect residual bias or instability. Therefore, the subsequent results regarding the moderating effect on this summary effect size should be interpreted with caution.

##### Moderating effects.

FA in SLF was significantly moderated by IQ (*b* = 0.05, *p* < 0.001), suggesting that differences in FA in SLF between ASD and TD decrease as IQ increases, while IQ did not moderate MD in SLF. Conversely, MD in SLF was significantly moderated by sex (*b* = −0.03, *p* = 0.037), suggesting that differences in MD in the SLF between ASD and TD reduce with a higher proportion of male participants, whereas sex did not moderate FA in SLF. Neither summary effect size was moderated by age or laterality (Table 1).

#### Uncinate Fasciculus

The uncinate fasciculus included 47 effect sizes for FA (*N*_*ASD*_ = 604, *N*_*TD*_ = 490) and 20 effect sizes for MD (*N*_*ASD*_ = 253, *N*_*TD*_ = 243).

##### Group differences.

**Figure 5:**
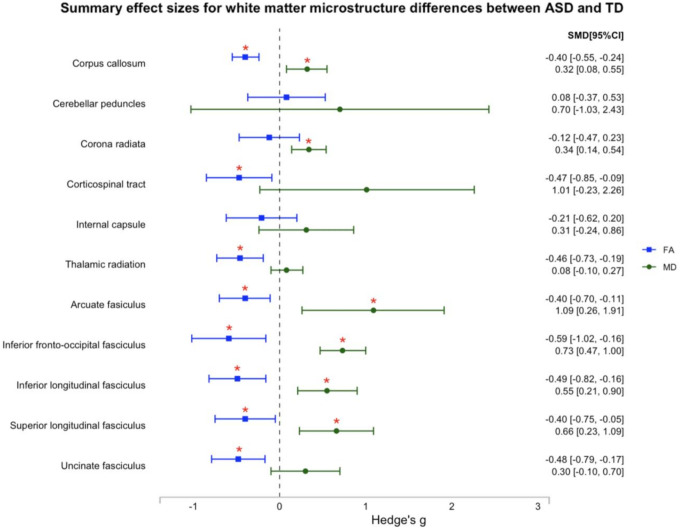
Illustration of all 11 summary effect sizes. *Note*. The effect sizes represent the revised estimates (after addressing influential cases), where relevant. * indicates a statistically significant summary effect size.

The analysis revealed a significant moderate summary effect size for FA in the uncinate fasciculus (*g* = −0.52, 95% CI [−0.83, −0.20], *p* = 0.001), indicating lower FA in the uncinate fasciculus in ASD compared to TD. In contrast, the summary effect size for MD in the uncinate fasciculus was non-significant (*g* = 0.31, 95% CI [−0.05, 0.67], *p* = 0.089). There was no evidence of small-study effects (FA: *b* = −2.75, 95% CI [−5.94, 0.45], *p* = 0.092; MD: *b* = 0.26, 95% CI [−3.79, 4.32], *p* = 0.899) or notable asymmetry in the funnel plots; however, several influential effect sizes were identified (*k*_*FA*_ = 2, *k*_*MD*_ = 4). The re-estimated summary effect sizes remained consistent in both magnitude classification and statistical inference for both FA (*g* = −0.48, *p* = 0.002) and MD (*g* = 0.30, *p* = 0.144) in the uncinate fasciculus. See Fig. 5 for an illustration of all 11 summary effect sizes.

##### Moderating effects.

FA in the uncinate fasciculus was significantly moderated by IQ (*b* = 0.06, *p* = 0.008), suggesting that differences in FA in the uncinate fasciculus between ASD and TD decrease as IQ increases, while IQ did not moderate MD in the uncinate fasciculus. Neither summary effect size was moderated by age, sex, or laterality (Table 1).

## Discussion

This meta-analysis quantified summary effect sizes for microstructural differences between ASD and TD groups across a wide range of white matter tracts. It also assessed how factors such as participant characteristics (age, sex, and IQ) and laterality contribute to the variability in these effect sizes. By focusing on FA and MD, the most consistently reported DTI metrics in ASD, we sought to clarify the anatomical distribution and robustness of white matter alterations associated with ASD.

### Altered White Matter Microstructure in ASD

Our meta-analysis revealed consistent white matter differences in individuals with ASD across FA and MD, particularly within long-range associative pathways. Reductions in FA were observed in the corpus callosum and all five examined association tracts (arcuate fasciculus, IFOF, ILF, SLF, and uncinate fasciculus), as well as in two of the five examined projection tracts (corticospinal tract and thalamic radiation). Similarly, increased MD was observed in the corpus callosum, corona radiata, and all association tracts except for the uncinate fasciculus. These findings align with prior meta-analyses and reviews [15, 22, 23], supporting the hypothesis that ASD is characterized by atypical long-range white matter connectivity, especially in tracts that facilitate higher-order social, linguistic, and cognitive processes [1, 3]. Notably, MD in the arcuate fasciculus and IFOF exhibited the most robust alterations, with large summary effect sizes, highlighting their potential role as key sites of microstructural disruption in ASD. These differences may emerge early in development; evidence from younger ASD samples indicates that microstructural alterations are present in infancy, suggesting disrupted connectivity as a core early feature of ASD neurodevelopment [18]. In contrast, the relatively limited and tract-specific involvement of projection pathways (corticospinal tract and thalamic radiation for FA; corona radiata for MD) and the absence of significant differences in tracts such as the cerebellar peduncles and internal capsule could suggest that lower-level sensorimotor and autonomic systems may be relatively preserved. Together, these findings reinforce the perspective that ASD is associated with distributed but tract-specific white matter alterations, disproportionately affecting networks responsible for higher-order integrative functions.

### Impact of Age, Sex, and Cognitive Ability on White Matter Microstructure in ASD Age

Our age-moderation analysis revealed that age moderates group differences in FA but not in MD, and only for a limited set of tracts. Specifically, FA differences in the corpus callosum, with lower values in individuals with ASD than in TD controls, were greater at older ages, consistent with findings suggesting that such alterations may emerge or intensify during adolescence and adulthood [15, 16, 36, 37]. In contrast, differences in FA in the IFOF and ILF, where FA is lower in individuals with ASD than in TD controls, were smaller at older ages, possibly reflecting maturational normalization or compensatory reorganization, although cross-sectional data limit the definitive interpretation. Prior meta-analytic evidence suggests that language-related tract differences are more prominent in children than in adults with ASD [23]. This pattern further aligns with findings of age-related normalization in language tracts [36] and broader age-by-diagnosis interactions across major white matter pathways in ASD [37]. Notably, no age-related moderation was observed in FA across projection tracts, nor in any tract for MD, despite prior evidence suggesting different age-related trajectories of MD in individuals with ASD compared to TD controls [20]. Together, these findings suggest that age may shape white matter differences in ASD in a regionally specific and FA-dominant manner. This underscores the need for longitudinal diffusion studies that follow changes within individuals throughout development and aging to advance our understanding of developmental neurobiology in ASD.

### Sex

Our sex-moderation analysis revealed that sex moderates group differences in a tract-specific manner. Across FA and MD, increased male representation in ASD samples was linked to diminished group differences in the corona radiata and arcuate fasciculus. For FA, this effect extended to thalamic radiation, while for MD, it encompassed the IFOF and SLF. Notably, FA in the corona radiata was moderated by sex despite the lack of a significant overall group difference, indicating that underlying differences between individuals with ASD and TD controls may depend on the sex composition of the samples. The tract-specific nature of these sex effects aligns with evidence that ASD involves sex-specific neurodevelopmental pathways, particularly in white matter organization [38]. Although some evidence suggests greater white matter divergence in males compared to females [39], our finding that higher male representation is associated with reduced ASD-TD differences may reflect dilution or masking of effects in more male-skewed samples (86% male in the ASD group, 82% male in the TD group). This interpretation is consistent with the ‘female protective effect’ and sex-differential liability models, which suggest that females require a higher genetic or environmental burden to manifest ASD traits [40]. The fact that only a subset of tracts demonstrated sex moderation underscores tract specificity, potentially reflecting the differential vulnerability of sensorimotor and language circuits to sex-linked mechanisms. This is supported by prior meta-analytic findings, which showed differences in language-related tracts such as the arcuate fasciculus, IFOF, and SLF, particularly in younger individuals with ASD, along with evidence that sex composition contributed to between-study heterogeneity [23]. In contrast, the absence of sex moderation in the commissural tract (i.e., corpus callosum) and most projection tracts may suggest more stable developmental profiles across sexes or insufficient variability in existing samples to detect interaction effects.

### IQ

Our IQ-moderation analysis revealed that IQ also moderates group differences in a tract-specific manner, having a more significant impact on FA than on MD. Specifically, differences in FA in the corticospinal tract, thalamic radiation, IFOF, ILF, SLF, and uncinate fasciculus diminished with higher IQ, suggesting a potential compensatory or adaptive mechanism that supports white matter development in these association and projection pathways. This pattern, however, contributes to the mixed findings regarding the association between IQ and white matter integrity in ASD. For example, Yeh et al. (2022) [41] reported white matter alterations were largely driven by individuals with ASD with co-occurring intellectual impairments, whereas those without intellectual impairment exhibited no significant differences from TD controls. In contrast, other studies have shown persistent FA reductions in individuals with ASD even after matching or covarying for IQ, especially in the anterior thalamic radiation, cingulum, arcuate fasciculus, and forceps minor [42, 43, 44]. Supporting this latter pattern, a tract-specific meta-analysis of language pathways [23] found no significant moderating effect of verbal IQ on diffusion differences in the arcuate fasciculus, IFOF, ILF, SLF, or uncinate fasciculus. Collectively, these findings suggest that while general cognitive ability may mitigate white matter alterations in some regions, certain neural differences are not fully accounted for by variability in either general cognitive ability or specific domains such as verbal IQ [23], highlighting the spatially distributed and heterogeneous nature of white matter alterations in ASD. Importantly, since IQ moderation was only partially assessed due to insufficient data for certain tracts, our findings may not provide a comprehensive account of how general cognitive ability interacts with white matter alterations in ASD.

### Laterality

Intriguingly, our meta-analysis revealed that laterality does not moderate group differences in most tracts across FA and MD, except for FA in the IFOF. Specifically, differences in FA were significantly greater in the left IFOF than in the right, indicating heightened vulnerability or disruption of left-lateralized white matter pathways in ASD. While this supports the hypothesis that certain left-lateralized language-related tracts may be especially vulnerable in ASD, it also contrasts with prior evidence of lateralization in cortical language regions [45]. Prior studies have also reported reduced or reversed hemispheric asymmetries in ASD, including in temporal and fronto-temporal regions [14, 46], and widespread reductions in cortical asymmetry, alongside increased putamen asymmetry, in ASD [46]. However, our finding that laterality effects emerged only in the IFOF diverges from these broader patterns, suggesting that atypical hemispheric organization in ASD may be more spatially selective than previously assumed, in line with meta-analytic evidence of tract-specific lateralization differences [23]. Alternatively, the absence of laterality effects in other tracts may reflect limited power to detect subtler asymmetries or variability in tract reconstruction across the studies included in our meta-analysis. Together, these findings reinforce the importance of hemispheric organization in ASD while emphasizing the need for more regionally targeted and harmonized investigations of lateralized white matter differences.

### Limitations

Several limitations must be acknowledged to contextualize the results. First, although we initially aimed to include multiple quantitative MRI techniques, such as CSD, DKI, NODDI, and relaxometry, the existing literature did not provide enough estimates per technique and outcome measure to support a robust meta-analysis. As a result, our analysis was limited to DTI. This methodological constraint highlights a broader gap in neuroimaging research in ASD, where advanced quantitative MRI techniques remain underutilized and minimally represented. While DTI offers exquisitely sensitive diffusion metrics that capture the magnitude and directionality of water movement in brain tissue, it is inherently non-specific and limited in resolving complex fiber geometries, capturing non-Gaussian diffusion, and differentiating between tissue compartments [47, 48]. Conversely, alternative techniques, such as CSD, DKI, NODDI, and relaxometry, provide more specific, biophysically informed parameters that complement and extend DTI findings. Broader application of these advanced techniques is necessary to deepen our understanding of microstructural alterations in ASD with improved specificity and sensitivity to underlying tissue characteristics.

Second, our meta-analysis was restricted to tracts with sufficient data. For the same reason, the analysis was confined to FA and MD due to limited data on other DTI metrics (AD and RD). Even among FA and MD, data availability varied considerably; MD estimates were available for only a few studies for some tracts. These limitations require cautious interpretation of the results for those regions. Additionally, although the tracts were generally categorized (e.g., corpus callosum, thalamic radiation), each encompasses distinct subregions. The lack of data for individual subregions (e.g., anterior, posterior vs. superior thalamic radiation) prevented us from exploring their potentially unique contributions to the observed effects. Variability in imaging analysis methods, such as ROI-based analysis, tractography techniques, and atlas use, may have also contributed to differences in the observed effects. However, due to high heterogeneity in analytic strategies, we could not reliably assess the imaging analysis method as a moderator. Like any meta-analysis, our study was limited by the data and reporting practices of the existing literature.

Sample characteristics also posed limitations. The samples included were primarily male, which limits the generalizability of our findings to females with ASD and prevents strong conclusions. Future research with a more balanced sex composition is essential to clarify sex-specific white matter differences in ASD. Furthermore, although age was considered as a moderator, this age range (2 to 50 years) excludes later adulthood, when white matter deterioration becomes more evident [49, 50]. Thus, our findings may not generalize across the entire lifespan, especially into older adulthood. Using age as a statistical moderator may also not adequately account for nonlinear or distinct biological developmental processes that influence brain microstructure at different life stages. Additionally, our cross-sectional data limit the ability to infer true developmental or aging-related changes within individuals. Meta-analyses of longitudinal studies are needed to determine whether observed age-related effects reflect within-subject trajectories rather than between-subject differences or cohort effects. Lastly, potential sample overlap between studies could not be eliminated due to limited transparency. While our model accounts for statistical dependence among multiple correlated effect sizes within studies, it does not explicitly address potential sample overlaps across different studies. Nonetheless, bias and sensitivity analyses under various statistical assumptions yielded consistent results, supporting the robustness of our conclusions.

## Conclusions

Our meta-analysis provides strong evidence of ASD-related alterations in white matter microstructure. It highlights tract-specific differences and the key role of several factors, such as age, sex, and general cognitive ability, in shaping the observed alterations. Overall, our findings underscore the multidimensional nature of white matter microstructure alterations in ASD, suggesting that these factors interact in intricate ways and contribute to variability in neurobiological patterns.

## Supplementary Files

This is a list of supplementary files associated with this preprint. Click to download.

- SupplementaryFiguresS1S3.docx
- SupplementaryTablesS1S3.docx

## Figures and Tables

**Table 1:** Results for the summary effect size of group differences and moderating effects (at *ρ* = 0.6), after addressing influential cases where relevant.

Tract	Summary effect size for group differences				Moderating effects											
					age (in years)			sex (% male)			IQ			laterality (left, right)		
	*g*	95% CI	*p*	*I* ^2^	*b*	95% CI	*p*	*b*	95% CI	*p*	*b*	95% CI	*p*	*b*	95% CI	*p*
**Fractional Anisotropy (FA)**																
Commissural Tract																
CC	− .40	[−.55, − .24]	**< .001**	44.0%	− .02	[−.03, − .002]	**.024**	.01	[−.01, .02]	.349	.002	[−.05, .05]	.949	-	-	-
Projection Tract																
CP	.08	[−.37, .53]	.731	80.4%	− .01	[−.05, .04]	.764	− .04	[−.15, .07]	.484	-	-	-	− .27	[−.83, .29]	.343
CR	− .12	[−.47, .23]	.494	78.1%	.01	[−.03, .06]	.592	.04	[.01, .08]	**.019**	.005	[−.06, .07]	.896	.001	[−.12, .12]	.988
CST	− .47	[−.85, − .09]	**.015**	77.7%	.03	[−.004, .07]	.078	.01	[−.001, .02]	.067	.03	[.001, .06]	**.039**	− .13	[−.29, .04]	.144
IC	− .21	[−.62, .20]	.306	77.1%	.02	[−.02, .07]	.264	.02	[−.03, .07]	.349	-	-	-	.16	[−.04, .35]	.112
TR	− .46	[−.73, − .19]	**< .001**	76.7%	.02	[−.01, .04]	.248	.01	[.0004, .02]	**.041**	.05	[.02, .08]	**.002**	.08	[−.32, .49]	.683
Association Tract																
AF	− .40	[−.70, − .11]	**.008**	36.6%	.02	[−.02, .06]	.238	.02	[−.0004, .04]	**.051**	-	-	-	.01	[−.20, .22]	.917
IFOF	− .59	[−1.02, − .16]	**.007**	80.2%	.06	[.01, .11]	**.013**	.01	[−.003, .03]	.099	.09	[.04, .13]	**< .001**	.20	[.04, .35]	**.012**
ILF	− .49	[−.82, − .16]	**.004**	74.6%	.04	[.01, .07]	**.007**	.01	[−.003, .02]	.145	.07	[.05, .10]	**< .001**	.06	[−.25, .36]	.724
SLF	− .40	[−.75, − .05]	**.027**	83.1%	.01	[−.03, .04]	.694	.01	[−.001, .03]	.072	.05	[.02, .08]	**< .001**	− .07	[−.31, .17]	.592
UF	− .48	[−.79, − .17]	**.002**	77.2%	.02	[−.02, .05]	.404	.004	[−.01, .02]	.593	.06	[.02, .10]	**.008**	.004	[−.18, .19]	.969
**Mean Diffusivity (MD)**																
Commissural Tract																
CC	.32	[.08, .55]	**.008**	63.2%	.01	[−.01, .03]	.163	− .01	[−.04, .01]	.343	-	-	-	-	-	-
Projection Tract																
CP	.70	[−1.03, 2.43]	.429	95.5%	− .03	[−.18, .12]	.683	.01	[−.19, .21]	.917	-	-	-	.05	[−.66, .76]	.887
CR	.34	[.14, .54]	**< .001**	0.00%	− .01	[−.02, .002]	.093	− .04	[−.07, − .01]	**.024**	.002	[−.04, .04]	.910	− .01	[−.23, .22]	.960
CST	1.01	[−.23, 2.26]	.111	92.9%	− .03	[−.16, .09]	.594	.01	[−.03, .06]	.618	− .001	[−.05, .05]	.958	− .04	[−.34, .26]	.804
IC	.31	[−.24, .86]	.275	78.7%	.02	[−.03, .06]	.432	− .02	[−.09, .05]	.553	-	-	-	− .08	[−.29, .12]	.424
TR	.08	[−.10, .27]	.382	11.5%	− .01	[−.03, .004]	.125	.01	[−.01, .03]	.389	− .01	[−.03, .01]	.249	.13	[−.09, .34]	.252
Association Tract																
AF	1.09	[.26, 1.91]	**.010**	85.3%	− .06	[−.17, .05]	.274	− .17	[−.27, − .08]	**< .001**	-	-	-	− .30	[−.79, .20]	.241
IFOF	.73	[.47, 1.00]	**< .001**	30.8%	.004	[−.03, .03]	.815	− .02	[−.03, − .001]	**.034**	.03	[−.02, .08]	.286	− .03	[−.34, .28]	.854
ILF	.55	[.21, .90]	**.002**	59.8%	.02	[−.01, .06]	.160	− .02	[−.04, − .001]	.067	− .03	[−.14, .09]	.623	− .13	[−.34, .07]	.203
SLF	.66	[.23, 1.09]	**.003**	75.0%	.02	[−.02, .05]	.442	− .03	[−.05, − .002]	**.037**	− .03	[−.07, .02]	.247	− .20	[−.59, .18]	.298
UF	.30	[−.10, .70]	.144	70.0%	− .01	[−.05, .04]	.785	− .02	[−.06, .01]	.243	.03	[−.02, .09]	.206	.03	[−.33, .39]	.875

*Note*. Negative effect size = a lower value in ASD compared to TD; positive effect size = a higher value in ASD compared to TD. - = not applicable; AC = arcuate fasciculus; CC = corpus callosum; CP = cerebellar peduncles; CR = corona radiata; CST = corticospinal tract; IC = internal capsule; IFOF = inferior fronto-occipital fasciculus; ILF = inferior longitudinal fasciculus; SLF = superior longitudinal fasciculus; TR = thalamic radiation.

## Data Availability

All data generated or analyzed during this study are available in the published articles included in this study and its supplementary information files.

## Abbreviations

AD: axial diffusivity
ASD: autism spectrum disorder
CSD: constrained spherical deconvolution
DKI: diffusion kurtosis imaging
DTI: diffusion tensor imaging
FA: fractional anisotropy
fMRI: functional magnetic resonance imaging
IFOF: inferior fronto-occipital fasciculus
ILF: inferior longitudinal fasciculus
MD: mean diffusivity
NODDI: neurite orientation dispersion and density imaging
PRISMA: Preferred Reporting Items for Systematic Reviews and Meta-Analyses Statement
RD: radial diffusivity
SD: standard deviation
SE: standard error
SLF: superior longitudinal fasciculus
TD: typically developing

## References

[1] Belmonte MK, Allen G, Beckel-Mitchener A, Boulanger LM, Carper RA, Webb SJ. Autism and abnormal development of brain connectivity. J Neurosci. 2004;24(42):9228–31. 15496656 10.1523/JNEUROSCI.3340-04.2004 PMC6730085

[2] Courchesne E, Pierce K. Why the frontal cortex in autism might be talking only to itself: local over-connectivity but long-distance disconnection. Curr Opin Neurobiol. 2005;15(2):225–30. 15831407 10.1016/j.conb.2005.03.001

[3] Just MA. Cortical activation and synchronization during sentence comprehension in high-functioning autism: evidence of underconnectivity. Brain. 2004;127(8):1811–21. 15215213 10.1093/brain/awh199

[4] Minshew NJ, Williams DL. The New neurobiology of autism: cortex, connectivity, and neuronal organization. Arch Neurol. 2007;64(7):945. 17620483 10.1001/archneur.64.7.945 PMC2597785

[5] Di Martino A, Yan CG, Li Q, Denio E, Castellanos FX, Alaerts K, The autism brain imaging data exchange: towards a large-scale evaluation of the intrinsic brain architecture in autism. Mol Psychiatry. 2014;19(6):659–67. 23774715 10.1038/mp.2013.78 PMC4162310

[6] Gotts SJ, Simmons WK, Milbury LA, Wallace GL, Cox RW, Martin A. Fractionation of social brain circuits in autism spectrum disorders. Brain. 2012;135(9):2711–25. 22791801 10.1093/brain/aws160 PMC3437021

[7] Uddin LQ, Supekar K, Lynch CJ, Khouzam A, Phillips J, Feinstein C, Salience network–based classification and prediction of symptom severity in children with autism. JAMA Psychiatry. 2013;70(8):869. 23803651 10.1001/jamapsychiatry.2013.104 PMC3951904

[8] Logothetis NK, Pauls J, Augath M, Trinath T, Oeltermann A. Neurophysiological investigation of the basis of the fMRI signal. Nature. 2001;412(6843):150–7. 11449264 10.1038/35084005

[9] Laumann TO, Snyder AZ, Mitra A, Gordon EM, Gratton C, Adeyemo B, On the stability of BOLD fMRI correlations. Cereb Cortex. 2016;27(10):4719–32. 10.1093/cercor/bhw265 PMC6248456 27591147

[10] Power JD, Barnes KA, Snyder AZ, Schlaggar BL, Petersen SE. Spurious but systematic correlations in functional connectivity MRI networks arise from subject motion. NeuroImage. 2012;59(3):2142–54. 22019881 10.1016/j.neuroimage.2011.10.018 PMC3254728

[11] Alexander AL, Lee JE, Lazar M, Field AS. Diffusion tensor imaging of the brain. Neurotherapeutics. 2007;4(3):316–29. 17599699 10.1016/j.nurt.2007.05.011 PMC2041910

[12] Alexander AL, Lee JE, Lazar M, Boudos R, DuBray MB, Oakes TR, Diffusion tensor imaging of the corpus callosum in autism. NeuroImage. 2007;34(1):61–73. 17023185 10.1016/j.neuroimage.2006.08.032

[13] Dean DC, Travers BG, Adluru N, Tromp DPM, Destiche DJ, Samsin D, Investigating the microstructural correlation of white matter in autism spectrum disorder. Brain Connect. 2016;6(5):415–33. 27021440 10.1089/brain.2015.0385 PMC4913512

[14] Lange N, DuBray MB, Lee JE, Froimowitz MP, Froehlich A, Adluru N, Atypical diffusion tensor hemispheric asymmetry in autism. Autism Res. 2010;3(6):350–8. 21182212 10.1002/aur.162 PMC3215255

[15] Travers BG, Adluru N, Ennis C, Tromp DPM, Destiche D, Doran S, Diffusion tensor imaging in autism spectrum disorder: a review. Autism Res. 2012;5(5):289–313. 22786754 10.1002/aur.1243 PMC3474893

[16] Travers BG, Tromp DPM, Adluru N, Lange N, Destiche D, Ennis C, Atypical development of white matter microstructure of the corpus callosum in males with autism: a longitudinal investigation. Mol Autism. 2015;6(1):15. 25774283 10.1186/s13229-015-0001-8 PMC4359536

[17] Ameis SH, Catani M. Altered white matter connectivity as a neural substrate for social impairment in autism spectrum disorder. Cortex. 2015;62:158–81. 25433958 10.1016/j.cortex.2014.10.014

[18] Faraji R, Ganji Z, Zamanpour SA, Nikparast F, Akbari-Lalimi H, Zare H. Impaired white matter integrity in infants and young children with autism spectrum disorder: what evidence does diffusion tensor imaging provide? Psychiatry Res Neuroimaging. 2023;335:111711. 37741094 10.1016/j.pscychresns.2023.111711

[19] Wang L, Ding S, Qin W, Zhang Y, Qin B, Huang K, Alterations in the white matter fiber tracts of preschool-aged children with autism spectrum disorder: an automated fiber quantification study. Quant Imaging Med Surg. 2024;14(12):9347–60. 39698649 10.21037/qims-24-950 PMC11652057

[20] McLaughlin K, Travers BG, Dadalko OI, Dean DC, Tromp D, Adluru N, Longitudinal development of thalamic and internal capsule microstructure in autism spectrum disorder. Autism Res. 2018;11(3):450–62. 29251836 10.1002/aur.1909 PMC5867209

[21] Jiang X, Shou XJ, Zhao Z, Chen Y, Meng FC, Le J, A brain structural connectivity biomarker for autism spectrum disorder diagnosis in early childhood. Psychoradiology. 2023;3:kkad005. 38666122 10.1093/psyrad/kkad005 PMC11003421

[22] Aoki Y, Abe O, Nippashi Y, Yamasue H. Comparison of white matter integrity between autism spectrum disorder subjects and typically developing individuals: a meta-analysis of diffusion tensor imaging tractography studies. Mol Autism. 2013;4(1):25. 23876131 10.1186/2040-2392-4-25 PMC3726469

[23] Li M, Wang Y, Tachibana M, Rahman S, Kagitani-Shimono K. Atypical structural connectivity of language networks in autism spectrum disorder: a meta-analysis of diffusion tensor imaging studies. Autism Res. 2022;15(9):1585–602. 35962721 10.1002/aur.2789 PMC9546367

[24] Zhang K, Fu Z, Lai Q, Zhao Y, Liu J, Cao Q. The shared white matter developmental trajectory anomalies of attention-deficit/hyperactivity disorder and autism spectrum disorders: a meta-analysis of diffusion tensor imaging studies. Prog Neuropsychopharmacol Biol Psychiatry. 2023;124:110731. 36764642 10.1016/j.pnpbp.2023.110731

[25] Zhao Y, Yang L, Gong G, Cao Q, Liu J. Identify aberrant white matter microstructure in ASD, ADHD and other neurodevelopmental disorders: a meta-analysis of diffusion tensor imaging studies. Prog Neuropsychopharmacol Biol Psychiatry. 2022;113:110477. 34798202 10.1016/j.pnpbp.2021.110477

[26] Page MJ, McKenzie JE, Bossuyt PM, Boutron I, Hoffmann TC, Mulrow CD, The PRISMA 2020 statement: an updated guideline for reporting systematic reviews. BMJ. 2021;372. 10.1136/bmj.n71 PMC8005924 33782057

[27] Hedges LV. Distribution theory for Glass’s estimator of effect size and related estimators. J Educ Stat. 1981;6(2):107–28.

[28] Wells G, Shea B, O’Connell D, Robertson J, Peterson J, Losos M, The Newcastle-Ottawa Scale (NOS) for assessing the quality of nonrandomized studies in meta-analyses. 2000.

[29] Assink M, Wibbelink CJM. Fitting three-level meta-analytic models in R: a step-by-step tutorial. Quant Methods Psychol. 2016;12(3):154–74.

[30] Cheung MWL. Modeling dependent effect sizes with three-level meta-analyses: a structural equation modeling approach. Psychol Methods. 2014;19(2):211–29. 23834422 10.1037/a0032968

[31] Viechtbauer W. Conducting meta-analyses in R with the metafor package. J Stat Softw. 2010;36:1–48.

[32] Nakagawa S, Santos ESA. Methodological issues and advances in biological meta-analysis. Evol Ecol. 2012;26(5):1253–74.

[33] Light RJ, Pillemer DB. Summing up: the science of reviewing research. Harvard University Press; 1984.

[34] Cook RD. Detection of influential observation in linear regression. Technometrics. 2000;42(1):65–8.

[35] Hunter JE, Schmidt FL. Methods of meta-analysis: correcting error and bias in research findings. Sage; 2004.

[36] Kleinhans NM, Pauley G, Richards T, Neuhaus E, Martin N, Corrigan NM, Age-related abnormalities in white matter microstructure in autism spectrum disorders. Brain Res. 2012;1479:1–16. 22902768 10.1016/j.brainres.2012.07.056 PMC3513400

[37] Weber CF, Lake EMR, Haider SP, Mozayan A, Mukherjee P, Scheinost D, Age-dependent white matter microstructural disintegrity in autism spectrum disorder. Front Neurosci. 2022;16:957018. 36161157 10.3389/fnins.2022.957018 PMC9490315

[38] Lai G, Pantazatos SP, Schneider H, Hirsch J. Neural systems for speech and song in autism. Brain J Neurol. 2012;135(3):961–75. 10.1093/brain/awr335 PMC3286324 22298195

[39] Zeestraten EA, Gudbrandsen MC, Daly E, De Schotten MT, Catani M, Dell’Acqua F, Sex differences in frontal lobe connectivity in adults with autism spectrum conditions. Transl Psychiatry. 2017;7(4):e1090–. 28398337 10.1038/tp.2017.9 PMC5416715

[40] Werling DM, Geschwind DH. Sex differences in autism spectrum disorders: Curr Opin Neurol. 2013;26(2):146–53. 23406909 10.1097/WCO.0b013e32835ee548 PMC4164392

[41] Yeh CH, Tseng RY, Ni HC, Cocchi L, Chang JC, Hsu MY, White matter microstructural and morphometric alterations in autism: implications for intellectual capabilities. Mol Autism. 2022;13(1):21. 35585645 10.1186/s13229-022-00499-1 PMC9118608

[42] Catani M, Dell’Acqua F, Budisavljevic S, Howells H, Thiebaut de Schotten M, Froudist-Walsh S, Frontal networks in adults with autism spectrum disorder. Brain J Neurol. 2016;139(2):616–30. 10.1093/brain/awv351 PMC4805089 26912520

[43] Haigh SM, Keller TA, Minshew NJ, Eack SM. Reduced white matter integrity and deficits in neuropsychological functioning in adults with autism spectrum disorder. Autism Res. 2020;13(5):702–14. 32073209 10.1002/aur.2271 PMC8237714

[44] Libero LE, DeRamus TP, Lahti AC, Deshpande G, Kana RK. Multimodal neuroimaging based classification of autism spectrum disorder using anatomical, neurochemical, and white matter correlates. Cortex. 2015;66:46–59. 25797658 10.1016/j.cortex.2015.02.008 PMC4782775

[45] Herbert MR, Harris GJ, Adrien KT, Ziegler DA, Makris N, Kennedy DN, Abnormal asymmetry in language association cortex in autism. Ann Neurol. 2002;52(5):588–96. 12402256 10.1002/ana.10349

[46] Postema MC, Van Rooij D, Anagnostou E, Arango C, Auzias G, Behrmann M, Altered structural brain asymmetry in autism spectrum disorder in a study of 54 datasets. Nat Commun. 2019;10(1):4958. 31673008 10.1038/s41467-019-13005-8 PMC6823355

[47] Jones DK, Knösche TR, Turner R. White matter integrity, fiber count, and other fallacies: The do’s and don’ts of diffusion MRI. NeuroImage. 2013;73:239–54. 22846632 10.1016/j.neuroimage.2012.06.081

[48] Jones DK, Cercignani M. Twenty-five pitfalls in the analysis of diffusion MRI data. NMR Biomed. 2010;23(7):803–20. 20886566 10.1002/nbm.1543

[49] Cox SR, Ritchie SJ, Tucker-Drob EM, Liewald DC, Hagenaars SP, Davies G, Ageing and brain white matter structure in 3,513 UK Biobank participants. Nat Commun. 2016;7(1):13629. 27976682 10.1038/ncomms13629 PMC5172385

[50] Sullivan EV, Pfefferbaum A. Diffusion tensor imaging and aging. Neurosci Biobehav Rev. 2006;30(6):749–61. 16887187 10.1016/j.neubiorev.2006.06.002

